# Detection of HPV DNA in Saliva of Patients with HPV-Associated Oropharyngeal Cancer Treated with Radiotherapy

**DOI:** 10.3390/curroncol31080328

**Published:** 2024-08-01

**Authors:** Atsushi Motegi, Shun-ichiro Kageyama, Yukie Kashima, Hidenari Hirata, Hidehiro Hojo, Masaki Nakamura, Takeshi Fujisawa, Tomohiro Enokida, Makoto Tahara, Kazuto Matsuura, Sadamoto Zenda

**Affiliations:** 1 Department of Radiation Oncology, National Cancer Center Hospital East, Kashiwa 277-8577, Japan; 2 Division of Radiation Oncology and Particle Therapy, Exploratory Oncology Research & Clinical Trial Center, National Cancer Center, Kashiwa 277-8577, Japan; 3 Department of Translational Informatics, Exploratory Oncology Research & Clinical Trial Center, National Cancer Center, Kashiwa 277-8577, Japan; 4 Department of Head and Neck Medical Oncology, National Cancer Center Hospital East, Kashiwa 277-8577, Japan; 5 Department of Head and Neck Surgery, National Cancer Center Hospital East, Kashiwa 277-8577, Japan

**Keywords:** Human-Papilloma-Virus (HPV), oropharyngeal cancer (OPC), saliva, chemoradiotherapy, cancer panel, biomarker

## Abstract

Background: To investigate the technical feasibility of RT–PCR and direct sequencing to quantify HPV DNA in the saliva of patients with Human-Papilloma-Virus related oropharyngeal cancer (HPV-OPC), the level of which is known to predict prognosis after treatment. Methods: Nine patients with locally advanced HPV-OPC treated with definitive radiotherapy with chemotherapy or cetuximab, or radiotherapy alone between April 2016 and September 2017, were enrolled, two of whom also received induction chemotherapy. Saliva was collected before (baseline), during (mid-RT) and after (post-RT) radiotherapy. HPV-16 DNAs (E6 and E7) in saliva were quantified by RT–PCR and sequencing, the latter using a custom cancer panel. Correlations between HPV DNA levels and clinical outcomes were assessed. Results: Compared to the baseline, the relative cycle threshold (Ct) value of E6 and E7 reduced at the point of mid-RT in the majority of the patients (100% and 75% for E6 and E7, respectively). Similarly, the relative Ct value from the baseline to post-RT reduced in 86% and 100% of the patients for E6 and E7, respectively. During the follow-up period, three patients (33%) experienced disease progression. The relative baseline Ct values of these three patients were in the top 4 of all the patients. The sequences of HPV DNA were detected in five (83%) of six samples of the baseline saliva that underwent DNA sequencing, along with several gene mutations, such as TP53,CDKN2A and PIK3CA. Conclusions: This study demonstrates that, in addition to detection and quantification of HPV DNA by RT–PCR, detection by sequencing of HPV-DNA using a customized cancer panel is technically possible.

## 1. Introduction

Radiotherapy alone or concurrent chemoradiotherapy is currently considered one of the standard of care for patients with HPV-OPC. Although patients with HPV-OPC who have small tumor volumes and a low smoking history are known to respond favorably to radiotherapy, a proportion of patients, approximately 15%, experience disease progression after treatment [1]. There are many clinical trials investigating the feasibility of de-escalating treatment intensity for patients with clinical backgrounds indicative of better prognosis [2,3]. Efforts are currently underway to discover novel biomarkers to further subclassify patients who are likely to respond better to these treatments [4].

A non-invasive diagnostic approach called liquid biopsy is attracting attention in clinical situations where tissue sampling is not easily performed [5]. Liquid biopsy, which uses body fluids such as plasma and saliva as a source of cancer specific DNA, has been shown to predict outcomes not only in patients with distant metastases but also in those with locally advanced disease [6,7]. Previous studies have shown that persistent detection of HPV DNA in saliva after radiotherapy correlates with disease recurrence and worse survival in patients with HPV-OPC [8]. However, it is still controversial as to whether the amount of HPV DNA in saliva collected before radiotherapy predicts treatment response and little is known how the amount of HPV DNA changes during radiotherapy. In addition, methods of HPV DNA detection have not been widely explored other than by RT–PCR despite the recent reduction in the cost of cancer panel sequencing [9,10]. Cancer panel sequencing can also detect cancer mutations, allowing a decision to be made as to whether or not a molecularly targeted drug is indicated [11].

We conducted a pilot study to assess the feasibility of detecting and quantifying HPV DNA alterations in the saliva from patients with HPV-OPC undergoing definitive radiotherapy throughout the treatment course (baseline, during and after treatment) using RT–PCR and with a custom cancer panel sequencing.

## 2. Materials and Methods

### 2.1. Study Population and Data Collection

This study was approved by the institute’s internal ethical review board (study number: 2016-353). Written informed consent was obtained from all patients enrolled in the study prior to enrollment. All patients had oropharyngeal cancer that was positive for p16 immunohistochemistry and were to undergo definitive radiotherapy using intensity-modulated radiotherapy (IMRT) with or without concurrent chemotherapy. Data on patient characteristics, treatment and outcomes were collected prospectively.

### 2.2. Inclusion and Exclusion Criteria

The inclusion criteria were as follows: patients (1) with histologically confirmed oropharyngeal squamous cell carcinoma, (2) who were scheduled to receive definitive radiotherapy, (3) who were over 20 years old, and (4) who agreed to participate in the study with a written informed consent document.

Patients deemed ineligible by the treating physician to be enrolled were excluded from the study.

### 2.3. Saliva Collection and DNA Extraction

Saliva was collected before (baseline), during (mid-RT), and after radiotherapy (post-RT) and stored at room temperature using Oragene (DNA Genotec, Stittsville, ON, Canada). When patients were to receive induction chemotherapy, saliva was collected before the start of induction chemotherapy. According to the schedule, mid-RT saliva was to be collected at the 4th week after the start of radiotherapy, and post-RT saliva was to be collected 60 days after the end of radiotherapy. DNA in saliva was extracted using DNeasy-and-Tissue Kit (Qiagen, San Diego, CA, USA) according to the manufacturer’s instructions. The quality of the extracted DNA was assessed using the Qubit assay kit (Thermo Fisher, Waltham, MA, USA).

### 2.4. Real-Time PCR

HPV DNA in saliva sample was quantified by RT–PCR using primers specifically designed to amplify HPV-16 E6 and E7. The sequences of each primer are as follows: E6 forward primer: 5′-TCAGGACCCACAGGAGCG-3′, E6 reverse primer: 5′-CCTCACGTCGCAGTAACTGTTG-3′, E7 forward primer: 5′-CCGGACAGAGCCCATTACAA-3′, E7 reverse primer: 5′-CGAATGTCTACGTGTGTGTGCTTTG-3′, beta-actin forward primer: 5′-TCACCCACACTGTGCCCATCTACGA-3′, beta-actin reverse primer: 5′-CAGCGGAACCGCTCAT- TGCCAATGG-3′. The amount of HPV DNA (E6 and E7) in saliva was analyzed comparing the relative cycle threshold (CT) values of the samples [12], where comparisons were made between all samples (irrespective of the time points and patients sampled) and within each sample (between the time points of the same patient). Standard curve for RT–PCR was generated using a dilution series constructed from a sample of HPV DNA which was arbitrarily selected. Beta-actin was used as a control.

### 2.5. Cancer Panel Sequencing

A custom cancer panel (Suredesign, ID: 3121311, Agilent, Santa Clara, CA, USA) was also designed to detect and quantify HPV-16 E6 and E7. Primers for HPV DNA by cancer panel sequencing were the same as those used for RT–PCR analysis. In addition, full exons of TP53, CDKN2A and PIK3CA were included in the cancer panel, as these are known to be the three most commonly mutated genes in head and neck squamous cell carcinoma [13]. A 50 ng DNA sample from each sample was indexed and the library was prepared as reported previously [14]. Following the capture of target regions using SureSelect DNA, samples were loaded onto Hiseq4000 (Illumina, San Diego, CA, USA) for sequencing. After data acquisition, mutation calling was performed using Agilent SureCall 4.1.2 software with default parameters. The minimum variant allele frequency (VAF) threshold was set at 0.001. Detection of HPV DNA by cancer panel sequencing was performed as follows: (1) fastq data were mapped to the reference data using mapping software (BWA version 0.7) after a necessary quality control process, (2) duplicates, unmapped reads and unmapped pairs were removed using Picard and samtools, (3) the ratio of HPV DNAs relative to the total sequence reads was calculated, followed by a quality control.

### 2.6. Statistical Analysis

In this study, a one-sided test with a *p*-value equal to or less than 0.05 was considered statistically significant. All statistical analysis was performed using JMP^®^ software version 10 (SAS, Tallahassee, FL, USA).

## 3. Results

### 3.1. Patient Characteristics

Table 1 shows patient characteristics. Saliva samples were collected from 9 patients with locally-advanced HPV OPC (Stage III–IV) between April 2016 and September 2017. These were 5 men and 4 women with a median age of 60 years (range: 44–74). All but one had primary tonsil disease. A total of 6 out of 9 patients had smoking history, with Brinkman index ranging from 0 to 900 (median: 90).

### 3.2. Treatment

All patients completed planned intensity-modulated radiotherapy, in which the gross disease (primary disease and lymph node metastasis) received 70 Gy and the entire lymph node area received 54–56 Gy as elective dose using simultaneous integrated boost technique over 7 weeks (daily, 33–35 fractions). There was no treatment interruption for more than 5 days in all patients.

Two patients received induction chemotherapy prior to concurrent chemoradiotherapy. Patient #1 received one cycle of TPS (docetaxel: 70 mg/m^2^ on days 1 and 8, cisplatin: 70 mg/m^2^ on day 4, and S-1: 100 mg/body on days 1–14) and then received radiotherapy alone because of renal dysfunction due to TPS. Patient #4 received seven cycles of PCE (paclitaxel: 80 mg/m^2^, cetuximab: initial dose of 400 mg/m^2^ followed by 250 mg/m^2^, carboplatin: AUC 1.5, weekly), then received RT with cetuximab (250 mg/m^2^, total seven cycles weekly) following PCE as local therapy. The remaining seven patients received chemoradiotherapy without induction chemotherapy; six patients received RT with cisplatin (20 mg/m^2^ on days 1–4, three cycles every three weeks), and another received RT with carboplatin and 5-FU (carboplatin AUC 5 on day 1 and 5-FU 500 mg/m^2^ on days 1–5, two cycles every four weeks).

### 3.3. Treatment Outcomes

**Figure 1:**
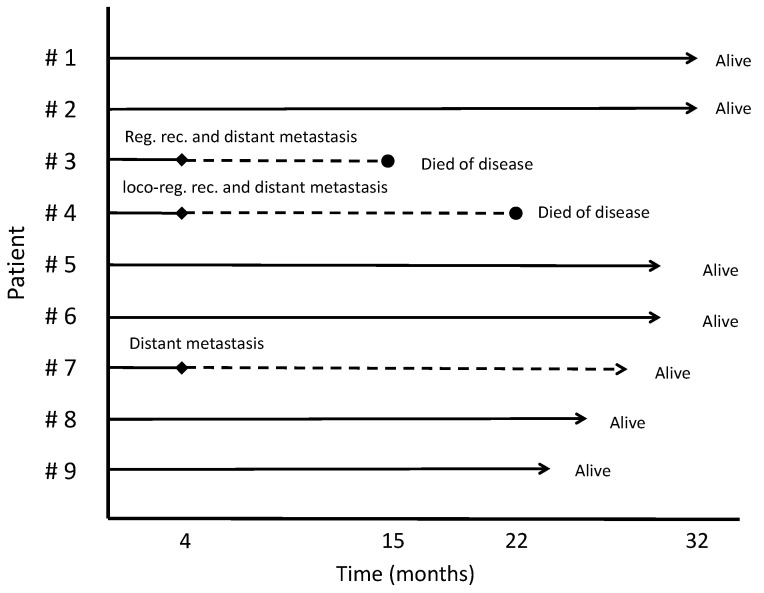
Diagram of the treatment outcomes. Abbreviation; Reg: regional. Loco-reg: loco-regional. Rhombus means any kind of recurrence. Dashed line indicates the period during which patients were alive recurrent disease. Circle means the time-point when patients deceased.

With a median follow-up of 32 months, 3 patients (33%) experienced local/regional recurrence or distant metastasis. Patient #3 had regional recurrence and distant metastasis 4 months after starting radiotherapy and died of the disease 15 months after starting radiotherapy. Patient #4 had local and regional recurrence and distant metastasis 4 months after starting radiotherapy and died of disease 22 months after starting radiotherapy. Patient #7 had a solitary distant metastasis at the lung 4 months after starting radiotherapy, which was surgically resected, and was alive at the last follow-up. Six patients (66%) were disease-free and alive at last follow-up. Figure 1 shows the chronological pattern of treatment outcomes for all patients.

### 3.4. Sample Collection and Quantification of Saliva HPV DNA by RT–PCR

**Figure 2:**
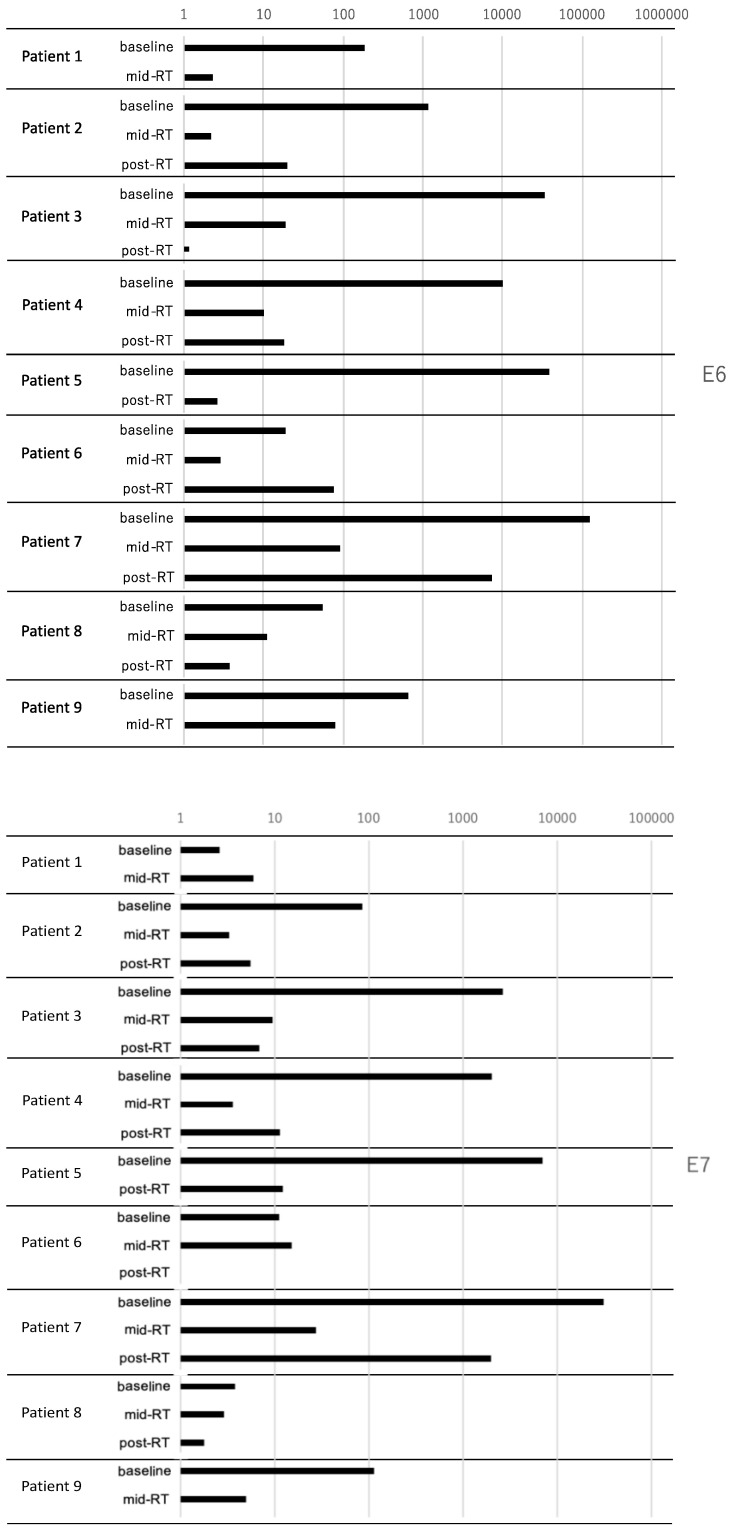
Relative Ct values of HPV DNA (E6 and E7) in saliva by RT–PCR.

Because several samples of saliva were not appropriately preserved due to technical problems, 24 saliva samples were collected in total. The baseline samples were collected from all patients, but mid-RT sample from patient #5, and post-RT samples from patients #1 and #9 were not collected. The range and median of the total DNA concentration from the samples were as follows: 48-1509 (620) ng/mL for baseline, 16-1929 (430) ng/mL for mid-RT, and 73-1120 (503) ng/mL. The relative CT values of HPV E6 and E7 at each time point are shown in Figure 2. From baseline to mid-RT, in which comparison was performed in 8 patients, the relative CT values for HPV E6 and E7 in saliva decreased in 8 (100%) and 6 (75%) patients, respectively. The median ratio of the relative CT value for HPV E6 and E7 in each patient from baseline to mid-RT were 0.0071 (range: 0.0006–0.19644) and 0.0409 (range: 0.0017–2.2686), respectively. From baseline to post-RT, in which comparison was performed in 7 patients, the relative CT values for HPV E6 and E7 in saliva similarly decreased 6 (86%) and 7 (100%) patients, respectively. The median ratio of the relative CT value for HPV E6 and E7 in each patient from baseline to post-RT were 0.0017 (range: 0.0001–4.1056) and 0.06353 (range: 0.0017–0.47600), respectively. However, the patterns of change of HPV E6 and E7 were not consistent from mid-RT to post-RT, in which comparison was performed in 6 patients, and decrease was observed in only 2 (33%) and 3 (50%) patients, respectively. The median ratios of the relative CT value for HPV E6 and E7 in each patient from mid-RT to post-RT were 5.4353 (range: 0.0528–82.3442) and 1.2048 (range: 0.72125–72.5547), respectively. 

When patients were sorted in order of the relative CT value for the baseline HPV DNA, the top 4 patients (i.e., those with higher baseline CT values: patient #7, #5, #3, and #4) were consistent for both HPV E6 and E7. Three patients (#3, #4, and #7), all of whom experienced recurrence 4 months after the start of radiotherapy, were among these top 4 patients (Table 2). Additionally, the correlation between the baseline relative CT values for HPV DNA and disease progression was examined. The Mann–Whitney U test showed marginal significance (both *p* = 0.070 for HPV E6 and E7) between the baseline relative CT values for HPV DNA and disease progression.

### 3.5. Cancer Panel Sequencing and HPV DNA Reads

Cancer panel sequencing was performed on 10 saliva samples from 6 patients, which were arbitrarily selected.

As shown in Table 3, HPV DNA was detected in 6 (67%) of 9 samples that underwent HPV DNA sequencing. When considering only the baseline samples, HPV DNA was detected in 5 (83%) of 6 samples. The range of HPV DNA reads was 2–16,377 (median: 1634 reads). HPV DNA reads were relatively high in samples from two patients who experienced recurrence, 1266 and 16,377 in patients #3 and #7, respectively.

Twenty-eight gene mutations were detected in TP53, PIK3CA and CDKN2A, of which five (18%) were nonsense mutations in PIK3CA and the rest were missense mutations.

## 4. Discussion

Although the number of patients and samples evaluated was small, this pilot study showed that HPV DNA was detectable and quantifiable in saliva samples from patients with p16-positive HPV-OPC treated with radiotherapy.

A recent review with meta-analysis reports that saliva-based circulating ctDNA testing would be acceptable for clinical use with an appropriate validation process [15]. Accordingly, several studies have shown that cancer-related genes are detectable in the saliva and plasma of patients with head and neck cancer [8]. Wang et al. showed that cancer-related DNAs (HPV DNAs detected by digital PCR in patients with HPV-OPC, and mainly p53 driver mutations in the others) were detectable in the saliva and plasma of patients with head and neck squamous cell carcinoma [14]. They also showed that patients with persistent or recurrent cancer-related DNA after treatment had worse outcomes. Hanna et al. used a highly sensitive digital droplet PCR assay to quantify HPV DNA in saliva and plasma [16]. They found that increased copy number of HPV DNAs in pre-RT saliva correlated with higher tumor burden and worse treatment response. Other reports on HPV detection in patients with OPC also suggest that saliva testing could be a reliable biomarker for predicting prognosis after treatment [17,18]. The current study is largely consistent with these studies, showing that three patients with relatively high HPV DNA Ct value in the baseline saliva developed progressive disease. In this study, it was also shown that HPV DNA (both E6 and E7) in the mid-RT saliva decreased greatly in most cases compared to the baseline. Even after excluding two patients who received induction chemotherapy, this finding remained. This indicates that the volume of primary tumor also decreased considerably at the time point around 40 Gy during the radiotherapy course.

We also showed that a customized cancer panel incorporating the HPV DNA sequence could detect HPV DNAs reads in the saliva. Although the detection rate of HPV DNAs was not as high as that by RT–PCR (100%), we could detect QC passed HPV reads from 5 (83%) of 6 baseline saliva samples along with several gene mutations, such as TP53, PIK3CA, and INK4A. To the best of our knowledge, this is the first technical report of a successful cancer panel incorporating viral DNA sequences.

In conclusion, we have shown that it is feasible to detect HPV DNA in the saliva from patients with HPV-OPC using RT–PCR and cancer panel sequencing. Based on the results of the current study, further research with a larger patient population is warranted to establish the role of saliva as a novel biomarker.

## 5. Conclusions

Detection and quantification of HPV DNA in saliva of patients with HPV-OPC by RT–PCR and sequencing were feasible. 

## Figures and Tables

**Table 1:** Patient characteristics. Abbreviation; m: male, f: female, BOT: base of tongue, conc.: concurrent, RT: radiotherapy, TPS: docetaxicel-cisplatin-S1, CBDCA: carboplatin, PCE: paclitaxel-carboplatin-cetuximab, Cmab: cetuximab.

Patient #	Stage (AJCC ver.7)	Sex	Age	Subsite	Brinkman Index	Chemotherapy
# 1	T2N2c	m	69	tonsil	800	TPS f/b RT alone
# 2	T4aN0	m	44	tonsil	345	conc. CDDP
# 3	T4aN2b	m	69	tonsil	800	conc. CBDCA
# 4	T4aN3	f	74	BOT	0	PCE f/b Conc.Cmab
# 5	T4aN2b	m	62	tonsil	672	conc. CDDP
# 6	T3N0	m	60	tonsil	90	conc. CDDP
# 7	T4aN2b	f	64	tonsil	0	conc. CDDP
# 8	T1N2b	f	66	tonsil	0	conc. CDDP
# 9	T2N2b	f	54	tonsil	50	conc. CDDP

**Table 2:** Relative quantity order of HPV E6 and E7 in the baseline saliva and presence/absence of Progressive Disease.

Patient #	Baseline Relative Quantity Order of		Progressive Disease
	HPV E6	HPV E7	
# 1	7	9	−
# 2	5	6	−
# 3	3	3	+
# 4	4	4	+
# 5	2	2	−
# 6	9	8	−
# 7	1	1	+
# 8	8	7	−
# 9	6	5	−

**Table 3:** List of the detected mutations and HPV DNA reads by cancer panel sequencing. Abbreviation; n/d: not detected, n/a: not available.

# of Patient	Timing of Collection	HPV DNA Reads	Gene	Type of Mutation
# 1	baseline	8	PIK3CA E707K TP53 G113D	MISSENSE MISSENSE
# 3	baseline	1266	PIK3CA A224T PIK3CA P421Q TP53 S212T TP53 G113D	MISSENSE MISSENSE MISSENSE MISSENSE
	Post-RT	n/d	PIK3CA G112V PIK3CA Q269K PIK3CA E418 PIK3CA W424L PIK3CA W1051 (tGf/tAg) PIK3CA W1051 (tgG/tgA) PIK3CA W1052K TP53 S212T	MISSENSE MISSENSE NONSENSE MISSENSE NONSENSE NONSENSE NONSENSE MISSENSE
# 4	baseline	4	PIK3CA E545K TP53 G113D	MISSENSE MISSENSE
	Post-RT	n/d	CDKN2A D23A CDKN2A E10G	MISSENSE MISSENSE
# 6	baseline	n/d	TP53 S212T	MISSENSE
	Post-RT	n/a	TP53 S212T	MISSENSE
# 7	baseline	16,377	PIK3CA E707K TP53 S212T	MISSENSE MISSENSE
# 8	baseline	1634	PIK3CAD84Y CDKN2AV25G	MISSENSE MISSENSE
	mid-RT	2	PIK3CA D84Y PIK3CA W1051 CDKN2A V25G TP53 E17K	MISSENSE NONSENSE MISSENSE MISSENSE

## Data Availability

The data that support the findings of this study are available on request from the corresponding author (A.M.).
